# Genomics and pharmacogenomics of cluster headache: implications for personalized management? A systematic review

**DOI:** 10.1097/YPG.0000000000000380

**Published:** 2024-11-12

**Authors:** Ulker Isayeva, Pasquale Paribello, Enrico Ginelli, Claudia Pisanu, Stefano Comai, Bernardo Carpiniello, Alessio Squassina, Mirko Manchia

**Affiliations:** a Unit of Psychiatry, Department of Medical Sciences and Public Health, University of Cagliari; b Unit of Clinical Psychiatry, Department of Medicine, University Hospital Agency of Cagliari; c Section of Neuroscience and Clinical Pharmacology, Department of Biomedical Sciences, University of Cagliari, Cagliari; d Division of Neuroscience, IRCCS San Raffaele Scientific Institute, Milan; e Department of Pharmaceutical and Pharmacological Sciences, University of Padua, Padua, Italy; f Neurobiological Psychiatry Unit, Department of Psychiatry, McGill University, Montreal, Quebec, Canada; g Department of Biomedical Sciences, University of Padua, Padua, Italy; h Department of Pharmacology, Dalhousie University, Halifax, Nova Scotia, Canada

**Keywords:** chronobiology, circadian rhythm, genetics, genome-wide association studies, lithium

## Abstract

The role of genetic factors in cluster headache etiology, suggested by familial and twin studies, remains ill-defined, with the exact pathophysiological mechanisms still largely elusive. This systematic review aims to synthesize current knowledge on cluster headache genetics and explore its implications for personalized treatment and prediction of treatment response. Thus, we searched PubMed, Scopus, and the Cochrane Library databases and reference lists of identified research articles, meta-analyses, and reviews to identify relevant studies up to 10 July 2024. The quality of the evidence was assessed using Newcastle-Ottawa Scale for case control studies and NIH Quality Assessment tool for Observational Cohort and Cross-Sectional Studies. The protocol of this study was registered via the Open Science Framework (https://osf.io/cd4s3). Fifty-one studies were selected for the qualitative synthesis: 34 candidate gene studies, 5 GWAS, 7 gene expression studies, 4 pharmacogenetic association studies, and 1 whole genome sequencing study. The bulk of genetic evidence in cluster headache underscores the involvement of genes associated with chronobiological regulation. The most studied gene in cluster headache is the *HCRTR2*, which is expressed in the hypothalamus; however, findings across studies continue to be inconclusive. Recent GWAS have uncovered novel risk loci for cluster headache, marking a significant advancement for the field. Nevertheless, there remains a need to investigate various genes involved in specific mechanisms and pathways.

## Introduction

Cluster headache is a devastating primary headache disease characterized by recurrent attacks of short-lasting excruciating pain accompanied by signs of autonomic dysfunction. Epidemiological studies have shown that cluster headache is likely to present prevalence at 1 person per 500, at least among people of European descent (Bjørn Russell, 2004). It has been shown that cluster headache is four times more frequent in men than women (Fischera *et al.*, 2008), however, in recent years this ratio has decreased with an increasing number of women being diagnosed with cluster headache (Steinberg *et al.*, 2019). Cluster headache is considered the most common form of trigeminal-autonomic cephalalgias (‘Headache Classification Committee of the International Headache Society (IHS) The International Classification of Headache Disorders, 3rd edition’, 2018). Cluster headache attacks are unilateral, severe, short-lasting (15–180 min), and recurrent (up to 8 attacks per day). The time during which recurrent attacks are occurring, usually weeks but at times months or years, is referred to as the cluster period. Cluster headache can be classified as episodic or chronic and is usually accompanied by ipsilateral cranial autonomic symptoms including lacrimation, nasal congestion, eyelid edema, forehead and facial sweating, conjunctival injection, miosis, and ptosis (Moreno-Ajona and Hoffmann, 2022). Additional symptoms of cluster headache are the sense of agitation or restlessness. Episodic cluster headache infers that pain-free periods, also known as remission periods, characterize the course of the disorder. Specifically, the attacks occur in period lasting from 7 days to 1 year separated by pain-free periods lasting for at least 3 months. Chronic cluster headache is characterized by attacks that occur for at least 1 year without remission or with limited remissions lasting less than 3 months. The chronic form of the disease can evolve from the episodic form (secondary chronic form) or may develop de novo as primary cluster headache (Dodick and Capobianco, 2001; Leroux and Ducros, 2008). It has been suggested that the factors such as alcohol consumption and smoking status might influence the transition from episodic to chronic form of cluster headache (Torelli *et al.*, 2000; Cho *et al.*, 2019). Moreover, smoking has been observed to be high in cluster headache population, with prevalence of 70% in females and 90% in males (Rozen, 2010; Chung *et al.*, 2021).

The exact pathophysiological mechanisms underlying cluster headache remain largely unclear. As per many complex neurological disorders, it is plausible to assume a multifactorial liability with genetics being a significant component of risk in interplay with environmental triggers. This theoretical framework has been partly supported by recent genomic analysis pointing to smoking intensity as a causal factor of cluster headache (Winsvold *et al*., 2023). Neurobiological mechanisms of cluster headache involve the complex interaction between the trigeminovascular system, the trigeminal autonomic reflex, and the hypothalamus. Hypothalamus plays a vital role in the regulation of the circadian rhythm, sleep-wake cycle, and neuroendocrine homeostasis (Settle, 2000; Saper and Lowell, 2014), all of which have shown to be associated with cluster headache (Leone *et al*., 2021). Many patients with cluster headache display circadian and circannual rhythmicity (Lee *et al.*, 2020) and the results of a recent meta-analysis showed a circadian pattern of attacks in 70% of participants with circannual peaks in spring and autumn and circadian peak between 21:00 and 03:00 (Benkli *et al.*, 2023). The temporal pattern associated with circadian and circannual rhythmicity of cluster headache suggests the dysfunctional regulation of biological clock in the pathophysiology of the disease (Naber *et al.*, 2019). The biological clock in mammals are regulated by the suprachiasmatic nucleus (SCN) which is located in the anterior hypothalamus, while hypocretin/orexin secreting neurons that are involved in sleep-wake cycle, feeding behavior, emotions, and pain processing are distributed in the posterior hypothalamus (Siegel, 2004). Patients with cluster headache have been found to exhibit reduced melatonin levels compared to healthy individuals (Chazot *et al.*, 1984; Leone *et al.*, 1995), suggesting the involvement of SCN in cluster headache, with the SCN being one of the most important targets of melatonin.

Albeit considered for many years a sporadic disease with absence of a heritable component, a number of twin and family studies indicated the existence of genetic factors underlying the susceptibility to cluster headache (Bjørn Russell, 2004; Russell, 2007; Montagna, 2008). Several studies assessing cluster headache in twins identified familial risk by reporting concordance among monozygotic twins (Roberge *et al.*, 1992; Sjaastad *et al.*, 1993; Schuh-Hofer *et al.*, 2003). Complex segregation analysis suggested an autosomal dominant mode of inheritance with low penetrance for cluster headache in some families and a multifactorial inheritance or autosomal recessive in other families (Russell *et al.*, 1995). The results of recent systematic review on family history of cluster headache showed that the positive family history rate of cluster headache varied between 0 and 22% with the median of 8.2% (Waung *et al.*, 2020). The study also examined the inheritance pattern of cluster headache across 67 pedigrees and found that most pedigrees were consistent with an autosomal dominant pattern in 69%, while 28% were consistent with an autosomal recessive pattern. The results of other meta-analysis evaluating the prevalence of familial cluster headache, showed a slightly lower prevalence rate of 6.27% (O’Connor *et al.*, 2020). Overall, first and second degree relatives show a higher likelihood of developing cluster headache compared to the general population (Leone *et al.*, 2001; Cruz *et al*., 2013) and although there is high variability in the estimated increased risk range across studies, these findings suggest an underlying hereditary factor in cluster headache. These studies have demonstrated the presence of familial loading for cluster headache and have prompted the analysis of molecular genetic determinants of risk for the disease.

Most existing research on genetic underpinnings of cluster headache focuses on candidate gene studies. However, these findings have often been inconsistent or difficult to replicate across different populations. This variability in reported results underscores the need for a systematic evaluation of the strength of genetic associations, assessment of replication across studies, and identification of potential areas for future research. This systematic review aims to address these gaps by synthesizing the current state of knowledge on the genetics of cluster headache, encompassing findings from targeted gene studies, gene expression studies, pharmacogenetic association studies, and recent genome-wide association studies (GWAS). By integrating these findings, the review will provide a clearer understanding of the genetic landscape and pathophysiology of cluster headache, with potential implications for personalized treatment. To the best of our knowledge, this is the first comprehensive systematic review to consolidate different types of genetic research on cluster headache, including both susceptibility risk and treatment outcomes, and to assess the quality of available evidence to help guide future research efforts.

## Methods

The protocol of this systematic review was registered via the Open Science Framework (https://osf.io/cd4s3) and followed the Preferred Reporting Items for Systematic Reviews and Meta-Analysis Protocols (PRISMA-P) 2020 guidelines (Page *et al.*, 2021) (Supplementary Figure 1, Supplemental digital content 1, http://links.lww.com/PG/A327).

### Search strategy

A systematic search was performed in PubMed, Scopus, and the Cochrane Library databases to identify relevant studies up to 10 July 2024. Search terms included a combination of phenotypic and genetic keywords and MeSH terms, such as ‘cluster headache’, ‘gene’, ‘genetic’, ‘genomic’, ‘genetic association’, ‘candidate gene’, ‘pharmacogenetic’, ‘pharmacogenomic’, ‘genome-wide association’, ‘GWAS’, ‘gene expression’, ‘polymorphism’, and ‘SNP’. No restrictions on publication date or language were applied. Additionally, reference lists of identified research articles, meta-analyses, and reviews were screened for further relevant studies. The search strategy was initially developed for PubMed and subsequently adapted for the other databases. The full search strategy is provided in Supplementary material, Supplemental digital content 1, http://links.lww.com/PG/A327.

### Eligibility criteria

Relevant articles were extracted using the following inclusion criteria: (a) studies that included patients diagnosed with cluster headache, (b) gene-expression studies, (c) candidate gene studies, (d) GWAS, and (e) pharmacogenetic association studies. Exclusion criteria were: nonhuman studies, non-English articles, review articles, book chapters, theses, studies without a genetic component (e.g. heritability or family studies), and non-peer-reviewed studies.

### Study selection

**Fig. 1:**
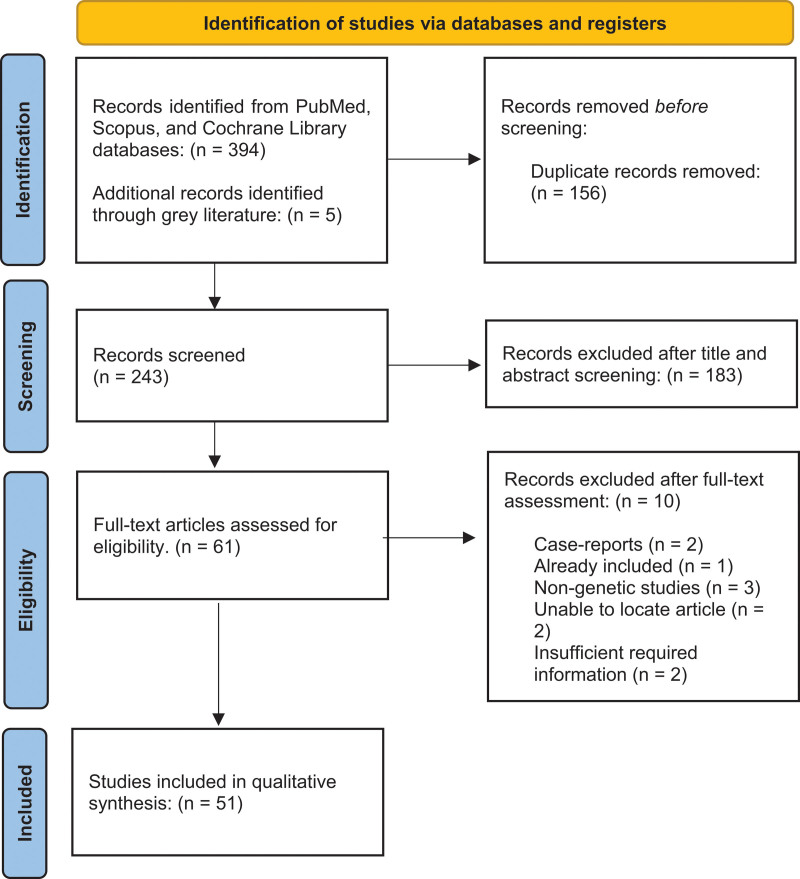
PRISMA flow diagram for the selection of studies. PRISMA, Preferred Reporting Items for Systematic Reviews and Meta-Analysis.

In the first stage, after removing duplicate records, two independent reviewers (U.I. and E.G.) blindly screened all eligible studies based on title and abstract using Rayyan (Ouzzani *et al.*, 2016). Full texts of potentially eligible studies that passed the initial screening were then evaluated by the same reviewers, and data were extracted using standardized forms. Any discrepancies were resolved through consensus, and if necessary, by consulting a third reviewer (P.P.). The detailed flowchart outlining the literature review process is presented in the PRISMA flowchart (Fig. 1).

### Data extraction and quality assessment

The following information was extracted from all included studies: first author’s name, publication year, country of study, study characteristics (sample size, study design, genotyping method, examined gene, and polymorphism), and main findings of the study. The quality of included studies were evaluated independently by two authors (U.I. and E.G.) using Newcastle-Ottawa Scale (NOS) for case-control studies (Wells *et al.*, 2000) and NIH quality assessment tools for observational cohort and cross-sectional studies (Study Quality Assessment Tools | NHLBI, NIH, 2024). The evaluation criteria of NOS scale allowed for a maximum score of 9 points. Articles were classified as high quality (7 or more points), moderate quality (4–6 points), and low quality (fewer than 4 points). For the NIH scale (with a total of 14 points), the thresholds for quality classification were as follows: ‘good’ (10–14 points), ‘fair’ (7–9 points), and ‘poor’ (0–6 points).

## Results

### Study selection

Through a comprehensive search across databases and grey literature, 399 records were initially retrieved, of which 156 were duplicates. After removing the duplicates, 192 articles were excluded as they did not meet the inclusion criteria. Ultimately, 51 studies met the inclusion criteria for the review, out of which 34 were candidate gene studies, 5 were GWAS, 7 were gene expression studies, 4 pharmacogenetic association studies, and 1 whole genome sequencing (WGS) study. The main characteristics of all included genetic association studies (candidate gene, WGS, and GWAS) are presented in Supplementary Table 1, Supplemental digital content 1, http://links.lww.com/PG/A327, while Supplementary Table 2, Supplemental digital content 1, http://links.lww.com/PG/A327 includes the characteristics of all included functional genomic and pharmacogenomic studies (gene expression and pharmacogenetic association studies).

### Study characteristics

The eligible studies were published between 1994 and 2024, consisting of 42 case-control (Gardiner *et al.*, 1998; Sjöstrand *et al.*, 2001, 2002, 2006; Rainero *et al.*, 2004, 2005, 2005, 2008, 2010; Baumber *et al.*, 2006; Schürks *et al.*, 2006, 2011; Cevoli *et al.*, 2008; Summ *et al.*, 2010; Steinberg *et al.*, 2011; Costa *et al.*, 2015; Weller *et al.*, 2015; Zarrilli *et al.*, 2015; Bacchelli *et al.*, 2016; Fourier *et al.*, 2016, 2018, 2019, 2021; Ofte *et al*., 2016; Eising *et al.*, 2017; Ran *et al.*, 2017, 2018, 2019; Fan *et al.*, 2018; Papasavva *et al.*, 2020; Harder *et al.*, 2021; Jennysdotter Olofsgård *et al.*, 2021, 2023, 2024; O’Connor *et al.*, 2021; Chen *et al.*, 2022; Papasavva, Vikelis, Katsarou, *et al.*, 2022; Papasavva, Vikelis, Siokas, *et al.*, 2022; Winsvold *et al*., 2023; Edvinsson *et al.*, 2024; Oliveira *et al.*, 2024; Petersen *et al.*, 2024), 4 cross-sectional (Shimomura *et al.*, 1994; Cortelli *et al.*, 1995; Seibel *et al.*, 1996; Petersen *et al.*, 2023), 3 cohort (Schürks, Kurth, Geissler, *et al.*, 2007; Schürks, Kurth, Stude, *et al.*, 2007; Schürks *et al.*, 2014), and 2 family studies (Haan *et al.*, 2001; Popescu, 2023).

### Genetic association studies

#### Targeted gene studies

The first candidate gene study on cluster headache involved the mitochondrial gene *MT-TL1* (*mtRNA^Leu(UUR)^*) with a 3243A>G point mutation that was identified in a single 52-year-old Japanese patient with episodic cluster headache (Shimomura *et al.*, 1994). However, subsequent studies attempting to replicate this finding have failed to confirm an association between this mitochondrial mutation and cluster headache. For instance, an Italian study involving 47 cluster headache patients (Cortelli *et al.*, 1995) and a German study with 22 cluster headache patients (Seibel *et al.*, 1996), found no evidence of the 3243 point tRNALeu(UUR) mutation, suggesting no significant link between this mutation and cluster headache.

The most studied gene in the susceptibility of cluster headache is *HCRTR2* gene that is expressed in the hypothalamus; however, results are not consistent across studies. Several studies have found a significant association between cluster headache and *HCRTR2*, specifically allele G of the G1246A polymorphism of the *HCRTR2* gene (Rainero *et al.*, 2004, 2008; Schürks *et al.*, 2006). Nonetheless, other studies (Baumber *et al.*, 2006; Weller *et al.*, 2015; Zarrilli *et al.*, 2015; Fan *et al.*, 2018; Fourier *et al.*, 2019; Papasavva *et al.*, 2020), did not support the association between G1246A polymorphism of the *HCRTR2* gene and the risk of cluster headache in diverse populations.

The rs1801260 polymorphism of the *CLOCK* gene has been reported to modify diurnal preference (Katzenberg *et al.*, 1998), however, so far no association has been identified between cluster headache and this SNP (Rainero *et al.*, 2005; Cevoli *et al.*, 2008; Zarrilli *et al.*, 2015; Fan *et al.*, 2018). A recent study also explored the association between cluster headache and rs1801260, as well as two other SNPs (rs11932595 and rs12649507) which had previously been linked to sleep duration (Allebrandt *et al.*, 2010), and reported a significant association with rs12649507 (Fourier *et al.*, 2018).

*PER1*, *PER2*, and *PER3* genes which are considered to be light sensitive clock genes (Sosniyenko *et al.*, 2009) have also been investigated in relation to cluster headache in a Swedish case-control study of 524 cluster headache cases and 680 controls (Jennysdotter Olofsgård *et al.*, 2021). Six *PER1*, *2*, and *3* genetic markers were genotyped for the purposes of the study, however, the results indicated no involvement of these genetic variants in cluster headache (Jennysdotter Olofsgård *et al.*, 2021). A variable number tandem repeat polymorphism of the *PER3* gene that has been previously associated with bipolar disorder (Brasil Rocha *et al.*, 2017) has also been investigated in a Norwegian cluster headache cohort, however no association was found (Ofte *et al*., 2016).

An Italian study of 110 patients with episodic and chronic cluster headache and 203 controls found a significant genetic association between *ADH4* SNP rs1126671, which has been associated with alcohol dependence and other addictive behaviors (Luo *et al.*, 2005; Preuss *et al.*, 2011), and cluster headache (Rainero *et al.*, 2010). Another Italian study explored the association between allele and genotype frequency of the rs1126671 and rs1800759 polymorphisms of the *ADH4* gene in cluster headache patients versus controls (Zarrilli *et al.*, 2015) finding significantly different allele and genotype frequency between sporadic cluster headache and controls. The attempt to further replicate the association between rs1800759 and rs1126671 polymorphisms of ADH4 and cluster headache failed in a large Swedish case-control cohort study of 390 cases and 389 controls (Fourier *et al.*, 2016). The associations were also not supported in a subsequent Chinese population based case-control study suggesting that rs1126671 and rs1800759 polymorphisms of *ADH4* gene are not genetic risk factors for cluster headache in the Chinese Han population (Fan *et al.*, 2018). Similarly, a recent study from Greece, failed to find an association between *ADH4* SNP rs1800759 and cluster headache (Papasavva *et al.*, 2020). They also examined the relationship between *GNB3* SNP rs5443 and cluster headache, but no significant association was reported (Papasavva *et al.*, 2020). In two subsequent targeted gene studies, the authors investigated the association of VDR gene polymorphisms rs2228570, rs1544410, and rs731236 with cluster headache susceptibility (Papasavva, Vikelis, Siokas, *et al.*, 2022), as well as the HFE H63D variant and cluster headache (Papasavva, Vikelis, Katsarou, *et al.*, 2022), but no associations were found. A study from Sweden that also examined the association between rs2228570, rs1544410, and rs731236 SNPs in the *VDR* gene and cluster headache susceptibility failed to report an association (Jennysdotter Olofsgård *et al.*, 2023). Additionally, an earlier study analyzing HFE gene polymorphisms C282Y and H63D in a cohort of 109 cluster headache patients and 210 controls likewise reported no significant associations (Rainero *et al.*, 2005).

The role of the *CACNA1A* gene in cluster headache was also explored due to its involvement in familial hemiplegic migraine and other neurological disorders. However, a Swedish study involving 75 cluster headache patients and 108 controls found no significant associations (Sjöstrand *et al.*, 2001). Additionally, a haplotype and mutation analysis of the *CACNA1A* gene was conducted in a family with three cluster headache-affected members, but no mutations were identified (Haan *et al.*, 2001). Similarly, the *MTHFR* gene which has been linked to migraine (Schürks *et al*., 2010) was not associated with cluster headache in a German cohort (Schürks *et al.*, 2011).

Ran and colleagues conducted several consecutive targeted gene studies investigating various genetic variants in relation to cluster headache. In the first study, they genotyped rs12668955 in *ADCYAP1R1*, rs1006417, an intergenic variant on chromosome 14q21, and one rare mutation, rs147564881 in *MME*, but found no significant association with cluster headache (Ran *et al.*, 2017). In a subsequent study, they found that rs1835740 in the *MTDH* gene significantly associated with cluster headache, while no association was found for the rs2651899 polymorphism in the *PRDM16* gene (Ran *et al.*, 2018). Lastly, they expanded their analysis to include *ANO3*, *ITGAL*, *PLCE1*, and *PCDHB6* genes and found rs1531394 in the ANO3 gene to be significantly associated with cluster headache. No significant associations were found for *ITGAL*, *PLCE1*, or *PCDHB6* (Ran et al., 2019).

One Swedish case-control study investigated the role of four SNPs within *CRY1* and *CRY2* genes in cluster headache; they selected rs2287161, rs8192440, rs10838524, and rs1554338 SNPs according to previous associations between them and several neurological and psychiatric disorders (Fourier *et al.*, 2021). They found that the variant rs8192440 in the *CRY1* gene was associated with cluster headache and the major allele G was more common in cluster headache patients than in controls. In a recent case-control study, Petersen et al. (2024) performed genotyping to assess shared genetic risk variants between cluster headache and testosterone concentrations in adult males and identified a shared genetic risk allele, rs112572874 in the *MAPT* gene. Lastly, there were two additional studies that examined the association of cluster headache with five polymorphic micro-satellite markers in the three different NO synthase (NOS) genes nNOS (NOS1), iNOS (NOS2A), and eNOS (NOS3) (Sjöstrand *et al.*, 2002) and SERPINA1 gene variants F, M, S, and Z (Summ *et al.*, 2010), but they did not detect any association.

#### Genome-wide association studies

In the five included GWAS, several polymorphisms were identified that were associated with susceptibility to cluster headache. An early Italian GWAS on 99 patients and 360 controls reported suggestive associations with a common variant of the PACAP receptor gene *ADCYAP1R1* (ADCYAP receptor type I) and *MME* (membrane metalloendopeptidase) (Bacchelli *et al.*, 2016); however it lacked statistical power and the reported associations were not replicated in a larger Swedish cohort (Ran *et al.*, 2017). Recently, there have been a few GWAS that showed a robust genetic association for cluster headache. A recent case-control GWAS that combined UK and Swedish cohorts involving 1443 cases and 6000 controls identified significant loci close to the genes that were indirectly involved with circadian rhythm (O’Connor *et al.*, 2021). The four cluster headache susceptibility loci that were identified by this GWAS were rs113658130 near *LINC01877/SATB2* (SATB homeobox 2), rs4519530 in *MERTK*, rs12121134 near *LINC01705/DUSP10* (Dual Specificity Phosphatase 10), and rs11153082 in *FHL5*. Another GWAS on individuals with European ancestry, specifically Dutch and Norwegian cohorts of 988 cases and 3257 controls combined, identified four independent loci associated with cluster headache (Harder *et al.*, 2021). The identified susceptibility loci were rs6541998 near *MERTK*, rs11579212 near *RP11-815 M8.1*, rs10184573 near *AC093590.1*, and rs2499799 near *UFL1/FHL5*, of which latter three were replicated in an independent sample. A GWAS conducted in a Taiwanese cohort involving 734 cases and 9846 controls identified three susceptibility loci with the SNPs being rs1556780 in *CAPN2*, rs10188640 in *MERTK*, and rs13028839 in *SATB2* (Chen *et al.*, 2022). Two of the identified loci (*MERTK* and *SATB2*) replicated the findings of the previous cluster headache GWAS conducted in European cohorts (Harder *et al.*, 2021; O’Connor *et al.*, 2021). The latest GWAS and meta-analysis on cluster headache obtained data from 10 European and 1 East-Asian cohort with a combined sample size of 4777 cases and 31 575 controls, and identified 8 loci of which 4 have been previously identified by GWAS (*DUSP10*, *MERTK*, *FTCDNL1*, and *FHL5*) that were also included in the meta-analysis (Winsvold *et al*., 2023).

#### Whole genome sequencing studies

In addition to GWAS and targeted gene approaches, one WGS study from France was also included in this review. The WGS on four members of the large multigenerational French family of cluster headache found that two family members showing the same phenotypic circadian pattern (familial periodicity) of symptoms had two genetic risk loci in the *HCRTR2* and in the *CLOCK* genes (Popescu, 2023). Thus, the risk of cluster headache appears to be significantly increased by the concomitant presence of these polymorphisms.

### Functional genomic and pharmacogenomic studies

#### Gene expression studies

Several studies have investigated the genetics of cluster headache using gene expression analysis approach. The study by Gardiner *et al*. (1998) showed that expression levels of α subunit of G-protein gene in lymphocytes were significantly reduced in cluster headache patients when compared with controls. The first microarray study performed in cluster headache (Sjöstrand *et al.*, 2006) found the upregulation of several S100 genes coding for calcium binding proteins during the active phase of the disease compared to remission, annexin A3 (calcium-binding), ICAM3, BIRC1 (neuronal apoptosis inhibitory protein), CREB5, and two human leukocyte antigen genes (*HLA-DQA1* and *HLA-DQB1*) that were upregulated in patients compared to controls. Furthermore, a microarray study that carried out whole transcriptome analysis in lymphoblastoid cell lines from 8 cluster headache and 10 bipolar disorder lithium responder patients found that two genes involved in the circadian system [RNA binding motif protein 3 (*RBM3*), and nuclear receptor subfamily 1, group D, member 1 (*NR1D1*)], were significantly dysregulated in both cluster headache and bipolar disorder patients when compared to controls (Costa *et al.*, 2015). Fourier *et al*. (2018) compared relative *CLOCK* gene expression levels between cluster headache cases and controls, and found no significant difference. However, they identified a significant increase in *CLOCK* mRNA expression when they investigated the effect of cluster headache associated rs12649507 SNP on differential *CLOCK* gene expression (Fourier *et al.*, 2018). In their other study, *CRY1* gene expression was slightly increased in cluster headache patients compared to controls, yet they could not confirm an effect of the cluster headache associated rs8192440 variant on general *CRY1* mRNA expression (Fourier *et al.*, 2021). Another study from Sweden found that T allele of cluster headache associated SNP rs1835740 had a significant effect on the transcriptional activity of *MTDH* gene, however, there was no difference in *MTDH* mRNA levels between cluster headache patients and controls (Ran *et al.*, 2018). In a subsequent study, they examined the cluster headache associated *ANO3* gene expression in patients and controls, but observed no difference between the two groups (Ran *et al.*, 2019). An additional study aimed to investigate cytokine interleukin-2 (IL-2) as a possible marker of immune system involvement in the pathophysiology of cluster headache. Patients with cluster headache showed an upregulation of the relative IL-2 gene expression during active cluster headache periods but not during attacks, remission, and in comparison to controls (Steinberg *et al.*, 2011). A study from Netherlands, obtained whole blood gene expression profiles of 39 patients with episodic and chronic cluster headache and 20 controls using RNA-seq approach and found no associations with genes involved in previously reported pathogenic mechanisms, including hypocretin dysregulation (Eising *et al.*, 2017). In a more recent study, relative *MERTK* gene expression was analyzed in 16 patients and 20 controls, showing increased *MERTK* mRNA levels in cluster headache patients. Additionally, elevated levels of the MERTK ligand Gal-3 were detected in serum samples of cluster headache patients compared to controls (Edvinsson *et al.*, 2024). Lastly, Oliveira *et al*. (2024) measured the CLOCK gene expression over multiple seasons, and the analysis showed a significant difference between patients and controls found in winter, spring, and summer, but not in autumn.

#### Pharmacogenetics of cluster headache

We included five pharmacogenetic association studies investigating the role of gene polymorphisms in drug responses in cluster headache. In an analysis of 184 cluster headache patients, no association between the *HCRTR2* G1246A polymorphism and treatment response to triptans, oxygen, verapamil, or corticosteroids was found (Schürks, Kurth, Geissler, *et al.*, 2007). Further analysis revealed that the chance of responding to a treatment with triptans for heterozygous carriers of the *GNB3* 825T allele was significantly increased when compared with homozygous carriers of the 825C allele (Schürks, Kurth, Stude, *et al.*, 2007). A more recent study by Schürks *et al*. (2014) examined the association between bi-allelic and tri-allelic *5-HTTLPR* genotypes and nonresponse to triptans, however the results were not statistically significant. Building on previous findings, three SNPs linked to verapamil response in migraine patients (Cutrer *et al.*, 2021) and four functional SNPs of liver enzymes *CYP3A4* were investigated for their association with verapamil treatment response in cluster headache patients; however, no significant association was found (Petersen *et al.*, 2023). In a most recent study, the association between five genetic variants rs1024905, rs6724624, rs4795541, rs5443, and rs2651899 and usage of triptans in cluster headache patients was analyzed. Results demonstrated that, rs1024905 was significantly associated with triptan non-usage in cluster headache (*Pc* = 0.010) (Jennysdotter Olofsgård *et al.*, 2024).

### Quality assessment of included studies

The methodological quality of the included studies varied considerably. Among the 42 case-control studies assessed using the NOS scale for case-control studies, scores ranged from 6 to 9, with a median score of 7 (Supplementary Table 3, Supplemental digital content 1, http://links.lww.com/PG/A327). Cross-sectional and cohort studies, assessed using the NIH quality assessment tools for observational cohort and cross-sectional studies, had scores ranging from 5 to 11, with a median score of 9 (Supplementary Table 4, Supplemental digital content 1, http://links.lww.com/PG/A327). In total, 32 studies were considered to be of high quality, 16 of medium quality, and 3 of low quality. The quality of the included studies was primarily affected by the lack of reporting on nonresponse rates during the recruitment process, absence of sample size justification or power description, and failure to adjust analyses for covariates and confounding variables.

## Discussion

In recent years, numerous studies have focused on identifying specific genetic risk factors for cluster headache, primarily employing a candidate gene approach. Several candidate genes for cluster headache have been investigated, including period circadian regulator 3 (*PER3*), circadian locomotor output cycles kaput (*CLOCK*), hypocretin receptor 2 (*HCRTR2*), calcium voltage-gated channel subunit alpha1 A (*CACNA1A*), alcohol dehydrogenase 4 (*ADH4*), nitric oxide synthase (*NOS*), and methylenetetrahydrofolate reductase gene (*MTHFR*). While family and twin studies suggest a genetic component of cluster headache, most candidate gene studies have failed to replicate early findings. The complex pathophysiology of cluster headache makes it challenging to identify genetic associations due to multiple factors including the interaction of multiple genes and the influence of various environmental risk factors. The circannual and circadian cyclicity of cluster headache symptoms, and the putative pathogenetic role for posterior hypothalamus strongly suggests that the circadian system cascade could be affected in cluster headache. Therefore, focusing on exploring the involvement of genes associated with chronobiological regulation and circadian rhythm may lead to deeper insights into the genetic underpinnings of cluster headache.

Given the presence of a substantial circadian variation in clinical symptoms, most candidate gene studies focused on components of the circadian cascade. This also motivated the investigation of the involvement of genes encoding for elements of the hypothalamus regulatory system including the orexinergic pathway. The hypocretins (orexins) are neuropeptides that are expressed in the lateral and posterolateral hypothalamus and are thought to play an important role in the regulation of arousal and the sleep/wake cycle (Date *et al.*, 1999). The hypocretinergic system is thought to be involved in the pathophysiology of cluster headache, particularly because hypocretin-containing neurons are located in the posterolateral hypothalamus which has been previously associated with cluster headache (Yang *et al.*, 2018; Buture *et al.*, 2019). The association between the G1246A polymorphism of the *HCRTR2* gene and cluster headache susceptibility has been extensively studied, with mixed results. Some studies have reported a significant association, while others have not. A recent meta-analysis (Yang *et al.*, 2020) also failed to support an association between the G1246A polymorphism of the *HCRTR2* gene and the overall risk of cluster headache in the population.

Alcohol is considered a common trigger during a cluster period (Rozen and Fishman, 2012) and it has been previously reported that those with chronic form of cluster headache tend to drink more alcohol than those with episodic cluster headache (Torelli *et al.*, 2000; Favier *et al*., 2005). Alcohol is metabolized by alcohol dehydrogenase and genetic variants within alcohol dehydrogenase 4 (*ADH4*) gene have been associated with alcohol and drug dependence. Therefore, *ADH4* gene which is located on chromosome 4q22-4q23 is of special interest in the pathophysiology of cluster headache. An examination of the data from included studies showed that the association between ADH4 and cluster headache risk was confirmed only in two Italian cohorts (Rainero *et al.*, 2010; Zarrilli *et al.*, 2015) but not in other populations (Fourier *et al.*, 2016; Fan *et al.*, 2018; Papasavva *et al.*, 2020). These discrepancies in the results could be explained by population differences between the cohorts or false positive results in the Italian studies given the limited sample sizes and likely inadequate power.

Several studies have investigated the role of the rs1801260 polymorphism of the circadian locomotor output cycles kaput (*CLOCK*) gene in cluster headache but did not report an association. *CLOCK* encodes a transcription factor that plays a key role in the function of the circadian system (Rainero *et al*., 2005) and is highly expressed in the SCN. One study reported a significant association between rs12649507 SNP in the *CLOCK* gene and cluster headache susceptibility and this association strengthened when stratified for reported diurnal rhythmicity of attacks (Fourier *et al.*, 2018). Beyond the *CLOCK* gene, the cryptochrome 1 and 2 (CRYs), brain and muscle ARNT-like 1 (BMAL1), and period circadian regulator 1, 2, and 3 (PERs) play a significant role in regulating circadian rhythms. These genes are integral components of the cellular clock, helping to maintain the synchronization of various physiological processes and are involved in the transcriptional/translational feedback loop that controls the expression of circadian genes throughout the day (Jin *et al.*, 1999; Lee *et al.*, 2010). Lithium,indicated as a prophylactic treatment for cluster headache has been found to significantly increase the expression of *Per2* and *Cry1*, and reduce the expression of *Per3*, *Cry2*, and *Bmal1* (Osland *et al.*, 2011). A recent meta-analysis reported that in three clinical trials with a total of 103 patients treated with lithium, 77% of the patients has either reached the composite outcome or reduced attack frequency by 50% (Pompilio *et al*., 2021). Lithium is an effective prophylactic agent for cluster headache (Bussone *et al.*, 1990; Dodick and Capobianco, 2001) and lithium responders with episodic course seem to exhibit association with genetic markers in HLA-B18 (Giacovazzo *et al.*, 1986). Lithium is also the mainstay of prophylactic treatment for bipolar disorder which is characterized by abnormalities in the circadian rhythms. In view of the direct in vitro effects of lithium on elements of circadian clock system (Yin *et al.*, 2006), the hypothesis of a possible implication of lithium targets as elements of the pathogenetic framework for cluster headache increased its strength. However, considering the limited number of studies on lithium in cluster headache, this suggestion needs to be explored in further research.

While reviewing the literature, we identified a notable lack of pharmacogenetic studies investigating the role of genetic variants in relation to treatment response in cluster headache. Several preventive treatments for cluster headache, including corticosteroids, triptans, melatonin, verapamil, valproic acid, and lithium, have also been shown to affect the circadian system. More recently, to evaluate whether polygenic risk scores (PRS) could aid in predicting the response to standard cluster headache treatments (oxygen, triptans, and verapamil), Petersen *et al*. (2023) conducted a study on 508 patients by genotyping several SNPs in CYP3A4 and applying PRS derived from a meta-analysis of the latest two cluster headache GWAS studies (Harder *et al.*, 2021; O’Connor *et al.*, 2021). The study found no significant effect of genetic variants or PRS in predicting treatment response (Petersen *et al.*, 2023). Other pharmacogenetic studies focused primarily on triptan treatment response in cluster headache, but their results have been inconclusive.

Based on the results of the included GWAS studies, *MERTK* appears to be a consistently identified genetic locus associated with cluster headache susceptibility. Multiple studies across different populations, including European cohorts (Harder *et al.*, 2021; O’Connor *et al.*, 2021; Winsvold *et al*., 2023) and a Taiwanese cohort (Chen *et al*., 2022), have replicated the association between variants near MERTK and cluster headache. *MERTK* is an interesting new candidate gene for cluster headache because it activates the cAMP-responsive element binding protein (CREB) and the CREB pathway has been implicated in timing and light entrainment of the SCN (Lee *et al.*, 2010). The replication of MERTK as a susceptibility locus strengthens the evidence for its involvement in the pathophysiology of cluster headache and highlights it as a promising candidate for further investigation. Additional findings of the latest GWAS included the genetic correlations of cluster headache with several traits including smoking, migraine, attention-deficit hyperactivity disorder, mood disorders, musculoskeletal pain, and risk-taking behavior (Winsvold *et al*., 2023).

The findings from gene expression studies in cluster headache provide valuable insights into the underlying molecular mechanisms, though they reveal a complex and inconsistent picture. The reduced expression of the G-protein α subunit (Gardiner *et al.*, 1998) and the upregulation of calcium-binding proteins, HLA genes, and apoptosis-related proteins during active cluster headache phases (Sjöstrand *et al.*, 2006) suggest a role for immune activation and low-grade inflammation in cluster headache pathophysiology. This notion is supported by Steinberg *et al*. (2011), who found upregulated IL-2 gene expression during active cluster headache periods. The dysregulation of circadian genes like RBM3 and NR1D1 in both cluster headache and bipolar disorder (Costa *et al.*, 2015) further strengthens the hypothesis of circadian system involvement, although subsequent studies, such as Fourier *et al*. (2018, 2021) showed mixed results regarding CLOCK and CRY1 gene expression. The discovery of increased MERTK mRNA levels and elevated Gal-3 in cluster headache patients (Edvinsson *et al.*, 2024) points out the potential role of this gene in cluster headache, further supported by previous GWAS studies that also identified MERTK as a susceptibility locus for the disorder.

The findings from gene expression studies in cluster headache provide valuable insights into the underlying molecular mechanisms, though they reveal a complex and inconsistent picture. The reduced expression of the G-protein α subunit (Gardiner *et al.*, 1998) and the upregulation of calcium-binding proteins, HLA genes, and apoptosis-related proteins during active cluster headache phases (Sjöstrand *et al.*, 2006) suggest a role for immune activation and low-grade inflammation in cluster headache pathophysiology. This notion is supported by Steinberg *et al*. (2011), who found upregulated IL-2 gene expression during active cluster headache periods. The dysregulation of circadian genes like RBM3 and NR1D1 in both cluster headache and bipolar disorder (Costa *et al.*, 2015) further strengthens the hypothesis of circadian system involvement, although subsequent studies, such as Fourier *et al*. (2018, 2021) showed mixed results regarding CLOCK and CRY1 gene expression. The discovery of increased MERTK mRNA levels and elevated Gal-3 in cluster headache patients (Edvinsson *et al.*, 2024) points out the potential role of this gene in cluster headache, further supported by previous GWAS studies that also identified MERTK as a susceptibility locus for the disorder.

This systematic review has several limitations that should be taken into consideration. Given the low prevalence of cluster headache, collecting adequately powered samples for large-scale genetic studies is challenging, resulting in limited sample sizes in the majority of the included studies. Despite evidence suggesting a role of genetic factors in cluster headache susceptibility and treatment response, very few studies have employed advanced genetic analysis techniques, and there is a complete absence of data at the epigenetic or proteomic levels. Approaches such as whole-exome sequencing and WGS could offer valuable insights into the molecular mechanisms underlying cluster headache susceptibility and the determinants of treatment response. These methodologies could enable the examination of a broader range of genes, including those involved in circadian regulation. Furthermore, while some genetic associations with cluster headache have been reported, most of these have been confirmed in only one or two studies, raising concerns about their robustness. Many of the included studies did not report a priori statistical power analyses, which increases the risk of both false negatives and overestimated effects. Future research should address these limitations by employing larger sample sizes, more comprehensive genetic approaches, and rigorous statistical validation.

One of the key strengths of this systematic review is its broad inclusion criteria, which encompass a variety of study types, including candidate gene studies, GWAS, gene expression, WGS, and pharmacogenetic studies. Furthermore, the use of rigorous methodological assessment tools, such as the NOS for case-control studies and the NIH quality assessment tool for observational studies, ensured that only methodologically sound studies were included in the analysis. As a result, this review provides a robust and reliable synthesis of the available evidence. To the best of our knowledge, this is the most extensive systematic review to date, offering a comprehensive qualitative synthesis of genetic association studies on cluster headache.

Evidence from clinical trials on cluster headache suggest differences in the underlying mechanisms of episodic and chronic cluster headache; therefore, stratification of cluster headache patients into subgroups according to their clinical traits may increase the efficiency of the future genetic research and help to identify additional susceptibility loci. In the absence of known causal pathways, it has been challenging to identify new therapeutic targets and predictors of treatment response. Making use of clinical features and biomarkers can facilitate the identification of patient-specific pathways, which could be essential for personalized management and improved clinical outcomes. Further progress into elucidating the genetic architecture of cluster headache can be achieved through larger studies using integrated analysis of genotypic and phenotypic factors.

## Acknowledgements

### Conflicts of interest

There are no conflicts of interest.

## Supplementary Material


