# Supplementary material for: Genomics and pharmacogenomics of cluster headache: implications for personalized management? A systematic review
Source: Psychiatr Genet. 2024 Nov 12;35(1):1–11. doi: 10.1097/YPG.0000000000000380 (PMC11698140; doi:10.1097/YPG.0000000000000380)
Supplement: Supplementary file 1 [file pg-35-1-s001.pdf]

Genomics and pharmacogenomics of cluster headache: implications for personalized management? A systematic review.

Supplementary Material

Table of Contents

Supplementary Figure 1: Checklist Summarizing Compliance with PRISMA Guidelines 2

Search strings used in the electronic databases 5

Supplementary Table 1: Summary of included genetic association studies 6

Supplementary Table 2: Summary of included functional genomic and pharmacogenomic studies 14

Supplementary Table 3. Quality assessment of case-control studies 17

Supplementary Table 4. Quality assessment of observational cohort and cross-sectional studies 19

Supplementary Figure 1: Checklist Summarizing Compliance with PRISMA Guidelines

Section and Topic	Item #	Checklist item	Location where item is reported
**TITLE**			
Title	1	Identify the report as a systematic review.	1
**ABSTRACT**			
Abstract	2	See the PRISMA 2020 for Abstracts checklist.	2
**INTRODUCTION**			
Rationale	3	Describe the rationale for the review in the context of existing knowledge.	4
Objectives	4	Provide an explicit statement of the objective(s) or question(s) the review addresses.	4
**METHODS**			
Eligibility criteria	5	Specify the inclusion and exclusion criteria for the review and how studies were grouped for the syntheses.	5
Information sources	6	Specify all databases, registers, websites, organisations, reference lists and other sources searched or consulted to identify studies. Specify the date when each source was last searched or consulted.	4
Search strategy	7	Present the full search strategies for all databases, registers and websites, including any filters and limits used.	5
Selection process	8	Specify the methods used to decide whether a study met the inclusion criteria of the review, including how many reviewers screened each record and each report retrieved, whether they worked independently, and if applicable, details of automation tools used in the process.	5
Data collection process	9	Specify the methods used to collect data from reports, including how many reviewers collected data from each report, whether they worked independently, any processes for obtaining or confirming data from study investigators, and if applicable, details of automation tools used in the process.	5
Data items	10a	List and define all outcomes for which data were sought. Specify whether all results that were compatible with each outcome domain in each study were sought (e.g. for all measures, time points, analyses), and if not, the methods used to decide which results to collect.	5
	10b	List and define all other variables for which data were sought (e.g. participant and intervention characteristics, funding sources). Describe any assumptions made about any missing or unclear information.	5
Study risk of bias assessment	11	Specify the methods used to assess risk of bias in the included studies, including details of the tool(s) used, how many reviewers assessed each study and whether they worked independently, and if applicable, details of automation tools used in the process.	5
Effect measures	12	Specify for each outcome the effect measure(s) (e.g. risk ratio, mean difference) used in the synthesis or presentation of results.	N/A
Synthesis methods	13a	Describe the processes used to decide which studies were eligible for each synthesis (e.g. tabulating the study intervention characteristics and comparing against the planned groups for each synthesis (item #5)).	5

Section and Topic	Item #	Checklist item	Location where item is reported
	13b	Describe any methods required to prepare the data for presentation or synthesis, such as handling of missing summary statistics, or data conversions.	N/A
	13c	Describe any methods used to tabulate or visually display results of individual studies and syntheses.	5
	13d	Describe any methods used to synthesize results and provide a rationale for the choice(s). If meta-analysis was performed, describe the model(s), method(s) to identify the presence and extent of statistical heterogeneity, and software package(s) used.	N/A
	13e	Describe any methods used to explore possible causes of heterogeneity among study results (e.g. subgroup analysis, meta-regression).	N/A
	13f	Describe any sensitivity analyses conducted to assess robustness of the synthesized results.	N/A
Reporting bias assessment	14	Describe any methods used to assess risk of bias due to missing results in a synthesis (arising from reporting biases).	N/A
Certainty assessment	15	Describe any methods used to assess certainty (or confidence) in the body of evidence for an outcome.	N/A
**RESULTS**			
Study selection	16a	Describe the results of the search and selection process, from the number of records identified in the search to the number of studies included in the review, ideally using a flow diagram.	5
	16b	Cite studies that might appear to meet the inclusion criteria, but which were excluded, and explain why they were excluded.	5
Study characteristics	17	Cite each included study and present its characteristics.	7
Risk of bias in studies	18	Present assessments of risk of bias for each included study.	Supplementary material
Results of individual studies	19	For all outcomes, present, for each study: (a) summary statistics for each group (where appropriate) and (b) an effect estimate and its precision (e.g. confidence/credible interval), ideally using structured tables or plots.	N/A
Results of syntheses	20a	For each synthesis, briefly summarise the characteristics and risk of bias among contributing studies.	10
	20b	Present results of all statistical syntheses conducted. If meta-analysis was done, present for each the summary estimate and its precision (e.g. confidence/credible interval) and measures of statistical heterogeneity. If comparing groups, describe the direction of the effect.	N/A
	20c	Present results of all investigations of possible causes of heterogeneity among study results.	9
	20d	Present results of all sensitivity analyses conducted to assess the robustness of the synthesized results.	N/A
Reporting biases	21	Present assessments of risk of bias due to missing results (arising from reporting biases) for each synthesis assessed.	N/A
Certainty of evidence	22	Present assessments of certainty (or confidence) in the body of evidence for each outcome assessed.	N/A

Section and Topic	Item #	Checklist item	Location where item is reported
**DISCUSSION**			
Discussion	23a	Provide a general interpretation of the results in the context of other evidence.	10
	23b	Discuss any limitations of the evidence included in the review.	13
	23c	Discuss any limitations of the review processes used.	13
	23d	Discuss implications of the results for practice, policy, and future research.	13
**OTHER INFORMATION**			
Registration and protocol	24a	Provide registration information for the review, including register name and registration number, or state that the review was not registered.	4
	24b	Indicate where the review protocol can be accessed, or state that a protocol was not prepared.	4
	24c	Describe and explain any amendments to information provided at registration or in the protocol.	4
Support	25	Describe sources of financial or non-financial support for the review, and the role of the funders or sponsors in the review.	14
Competing interests	26	Declare any competing interests of review authors.	14
Availability of data, code and other materials	27	Report which of the following are publicly available and where they can be found: template data collection forms; data extracted from included studies; data used for all analyses; analytic code; any other materials used in the review.	14

Search strings used in the electronic databases:

PubMed
((((((("Cluster Headache/genetics"[Mesh])) OR "cluster headache"[Title/Abstract] OR "cluster headaches"[Title/Abstract])) AND ("Genetics"[Mesh] OR "genetic"[Title/Abstract] OR "genomic"[Title/Abstract] OR "gene"[Title/Abstract])) OR ("Genetic Association Studies"[Mesh] OR "genetic association"[Title/Abstract] OR "candidate gene"[Title/Abstract])) OR ("Genome-Wide Association Study"[Mesh] OR "GWAS"[Title/Abstract] OR "genome-wide association"[Title/Abstract])) OR ("Gene Expression"[Mesh] OR "gene expression"[Title/Abstract])) OR ("Pharmacogenetics"[Mesh] OR "pharmacogenetic"[Title/Abstract] OR "pharmacogenomic"[Title/Abstract])) OR ("Polymorphism, Genetic"[Mesh] OR "polymorphism"[Title/Abstract] OR "SNP"[Title/Abstract]))

Cochrane Library
(((((((([mh "Cluster Headache"])) OR "cluster headache" OR "cluster headaches")) AND ([mh Genetics] OR genetic OR genomic OR gene))
OR ([mh "Genetic Association Studies"] OR "genetic association" OR "candidate gene"))
OR ([mh "Genome-Wide Association Study"] OR GWAS OR "genome-wide association"))
OR ([mh "Gene Expression"] OR "gene expression")) OR ([mh Pharmacogenetics] OR pharmacogenetic OR pharmacogenomic)OR ([mh "Polymorphism, Genetic"] OR polymorphism OR SNP)

Scopus
((((((("Cluster Headache")) OR "cluster headache" OR "cluster headaches")) AND (Genetics OR genetic OR genomic OR gene))
OR ("Genetic Association Studies" OR "genetic association" OR "candidate gene"))
OR ("Genome-Wide Association Study" OR GWAS OR "genome-wide association"))
OR ("Gene Expression" OR "gene expression")) OR (Pharmacogenetics OR pharmacogenetic OR pharmacogenomic) OR ("Polymorphism, Genetic" OR polymorphism OR SNP

Supplementary Table 1. Summary of included genetic association studies

Reference	Country	Study Type	Sample	Methods	Genes Examined	Result highlights
**Bacchelli et al., 2016**	Italy	GWAS	99 CH, 360 controls	Genotype data were used to carry out a genome-wide single marker case-control association analysis using common SNPs, and a gene-based association analysis focusing on rare protein altering variants in 745 candidate genes with a putative role in CH.	1038 genes	Associations with a common variant of the PACAP receptor gene *ADCYAP1R1* (P = 9.1 × 10−6) and *MME* (P = 2.5 × 10−5)
**Baumber et al., 2006**	Denmark, Italy, Sweden, UK	Candidate gene	33 families and 62 individuals genotyped, 270 cases and 267 controls	Genomic DNA was extracted, followed by PCR amplification and genotyping of microsatellite markers and SNPs, with subsequent sequencing of the HCRTR2 gene for variant detection.	*HCRTR2*	No association and no deleterious sequence variants of the HCRTR2 gene were detected. Potential linkage was identified at four disease loci: two distinct loci on chromosome 2 (markers D2S1353; 164.51 cM, and D2S1363; 227.0 cM), and one locus on each of chromosome 8 (GATA151F02; 27.4 cM) and chromosome 9 (D9S2169; 14.23 cM)
**Cevoli et al., 2008**	Italy	Candidate gene	101 CH (85 episodic and 16 chronic CH), 100 controls	T3111C CLOCK polymorphism (also known as T3092C) was genotyped in patients and controls by enzymatic digestion with Sdu I (Fermentas) and electrophoresed on MetaPhor agarose 2 % gel	*CLOCK*	No association found
**Chen et al., 2022**	Taiwan	GWAS	734 CH, 9846 controls	A two-stage GWAS in a Taiwanese cohort enrolled from 2007 through 2022 to identify the genetic variants associated with CH. Downstream analyses including gene-set and tissue enrichment, linkage disequilibrium score regression, and pathway analyses were performed.	*CAPN2, MERTK, STAB2*	Three replicable loci, with the lead SNPs being rs1556780 in CAPN2 (odds ratio=1.59, 95% CI 1.42–1.78, p=7.61× 10–16), rs10188640 in MERTK (odds ratio=1.52, 95% CI 1.33–1.73, p=8.58× 10–13), and rs13028839 in STAB2 (odds ratio=0.63, 95% CI 0.52–0.78, p=2.81× 10–8), with the latter two replicating the findings in European populations
**Cortelli et al., 1995**	Italy	Candidate gene	47 CH cases	Analysis of mitochondrial	*mtRNALeu(UUR)*	No mutations found

				tRNA Leu(UUR) 3243A>G point mutation in 47 patients affected by CH (blood cells)		
**Fan et al., 2018**	China	Candidate gene	112 CH patients and 192 controls	Genomic DNA was extracted from lymphocytes using the TIANamp Blood DNA Kit, and SNP genotyping was performed using MassARRAY Analyzer 4 System	*HCRTR2, ADH4 and CLOCK*	No association between HCRTR2, ADH4, CLOCK gene polymorphisms and CH was found, however, haplotype analysis indicated H1-GTGGGG was linked to a reduced CH risk (44.7% vs. 53.1%, OR = 0.689, 95% CI =0.491~0.966, p = 0.030)
**Fourier et al., 2016**	Sweden	Candidate gene	390 CH, 389 controls	DNA samples from patients and controls were genotyped for the two ADH4 SNPs rs1126671 and rs1800759 using quantitative real-time PCR.	*ADH4*	No association found
**Fourier et al., 2018**	Sweden	Candidate gene and gene expression study*	447 CH, 677 controls	Genotyping of rs1801260, rs11932595, and rs12649507 SNPs of CLOCK gene was performed using quantitative Real-Time PCR (qPCR). For gene expression, fibroblast cultures were established from biopsies of 11 CH patients and 11 controls, synchronized with serum treatment, and RNA was extracted using the QIAGEN RNeasy Mini Kit	*CLOCK*	Significant association with rs12649507 and CH ($p = 0.0069$) was found. No difference in *CLOCK* mRNA expression between patients and controls. A significant effect of rs12649507 on *CLOCK* gene expression in human primary fibroblast cultures was found ($p = 0.0232$)
**Fourier et al., 2019**	Sweden	Candidate gene	517 CH, 581 controls	Genotyped rs3122156, rs2653342, and rs2653349 SNPs in the HCRTR2 gene using quantitative real-time PCR	*HCRTR2*	No association found
**Fourier et al., 2021**	Sweden	Candidate gene and gene expression study*	628 CH, 681 controls	Four known genetic variants in the CRY1 (rs2287161 and rs8192440) and CRY2 (rs10838524 and rs1554338) genes were genotyped in all patients and controls. CRY1 gene expression in primary fibroblast cell lines was analyzed in 11 patients and 10 controls	*CRY1, CRY2*	The exonic CRY1 variant rs8192440 was associated with CH on allelic level (p=0.02) and this association was even more pronounced in patients with reported diurnal rhythmicity of attacks (p=0.002). Significant difference in CRY1 gene expression between CH patients and controls (p=0.04) was found.

**Haan et al., 2001**	Netherlands	Candidate gene	3 family members (1 proband, 1 daughter with CH, 1 cousin with CH)	Genomic DNA isolated from leucocytes; Haplotype analysis using microsatellite markers on chromosome 19; PCR for all markers, followed by SSCP analysis of all 47 exons to identify mutations.	*CACNA1A* (47 exons)	No mutations found in the CACNA1A gene.
**Harder et al., 2021**	Netherlands, Norway	GWAS	988 CH, 3257 controls	Case-control GWAS. Gene set and tissue enrichment analyses, blood cell-derived RNA-sequencing of genes around the risk loci and linkage disequilibrium score regression was part of the downstream analyses.	*RP11-815 M8.1, MERTK, AC093590.1, UFL1/FHL5*	An association was found with CH for 4 independent loci (r2 < 0.1) with genome wide significance ($p < 5 \times 10^{-8}$), rs11579212 near RP11-815 M8.1, rs6541998 near MERTK, rs10184573 near AC093590.1, and rs2499799 near UFL1/FHL5. Gene-based mapping yielded ASZ1 as possible fifth locus. RNA-sequencing indicated differential expression of POLR1B and TMEM87B in CH patients
**Jennysdotter Olofsgård et al., 2021**	Sweden	Candidate gene	524 CH, 680 controls	Six PER1, 2 and 3 genetic markers; the indel rs57875989 and five SNPs, rs2735611, rs2304672, rs934945, rs10462020, and rs228697, were genotyped, using TaqMan® or regular polymerase chain reaction (PCR)	*Per1, Per2, Per3*	No association found
**Jennysdotter Olofsgård et al., 2023**	Sweden	Candidate gene	617 CH patients and 672 controls	Genotyping for rs2228570 was performed in Swedish participants using qPCR; rs1544410 and rs731236 genotyping data were obtained from previous GWAS.	*VDR*	No significant association was found between CH and the three SNPs.
**O'Connor et al., 2021**	Sweden, UK	GWAS	1443 CH, 6000 controls	Case-control GWAS Downstream analyses, such as gene-set enrichment, functional variant annotation, prediction and pathway analyses, were performed.	*LINC01877/SATB2, MERTK, LINC01705/DUSP10, FHL5*	The lead SNPs were rs113658130 ($p = 1.92 \times 10^{-17}$, odds ratio [OR] = 1.51) and rs4519530 ($p = 6.98 \times 10^{-17}$, OR = 1.47) on chromosome 2, rs12121134 on chromosome 1 ($p = 1.66 \times 10^{-8}$, OR = 1.36), and rs11153082 ($p = 1.85 \times 10^{-8}$, OR = 1.30) on chromosome 6

**Ofte et al., 2016**	Norway	Candidate gene	149 CH, 432 controls	Genotyping of PER3 VNTR was performed using PCR amplification with specific primers followed by gel electrophoresis to differentiate genotypes in patients controls	*Per3*	No association found
**Papasavva et al., 2020**	Greece	Candidate gene	114 CH, 570 controls	rs2653349, rs5443, and rs1800759 polymorphisms of HCRTR2, GNB3, and ADH4 genes were genotyped using real-time PCR	*HCRTR2, GNB3, and ADH4*	No association found
**Papasavva et al., 2022a**	Greece	Candidate gene	128 CH, 294 controls	Genotyping using SNP TaqMan qPCR from buccal swabs. Analysis of genotypic and allelic frequency, and subgroup analysis for episodic and chronic CH.	*HFE*	No significant association found between the HFE H63D variant and CH susceptibility overall. CC genotype and C allele frequencies were significantly higher in patients with episodic compared to chronic CH patients (OR [95% CI] 2.428 [1.055–5.584], $p = 0.034$ dominant genotypic model and OR [95% CI] 2.161 [1.059–4.410], $p = 0.032$ allelic model).
**Papasavva et al., 2022b**	Greece	Candidate gene	131 CH, 281 controls	Genotyping of *VDR* gene polymorphisms (*FokI*-rs2228570, *BsmI*-rs1544410, and *TaqI*-rs731236) performed using real-time PCR and melting curve analysis.	*VDR*	No significant association between VDR polymorphisms and CH susceptibility. A more frequent occurrence of CH attacks (≥ 4 attacks/day) was observed in patients carrying the *BsmI* GG genotype (GG vs. GA + AA: OR 0.343, 95% CI 0.144–0.816, $P = 0.016$). TAC haplotype associated with decreased CH risk (OR 0.634, 95% CI 0.400–1.004, $P = 0.05$).
**Petersen et al., 2024**	Denmark	Candidate gene	60 males with episodic CH, 60 males with chronic CH, 60 controls.	Genotyping was performed to assess shared genetic risk variants using data from GWAS for testosterone levels and CH.	*MAPT*	A shared genetic risk allele, rs112572874 (in the intron of the MAPT gene), was identified between fT and CH, suggesting a pathophysiological link between CH and testosterone. Reduced fT/LH ratio in patients with chronic CH by 35% and episodic CH by 24% compared to controls.

**Popescu et al., 2023**	France	Whole genome sequencing	4 patients with CH	Whole genome sequencing (WGS) was performed in four members of the multigenerational family of CH	*HCRTR2, CLOCK*	Combination of *HCRTR2* and *CLOCK* gene polymorphisms in 2 members of the family was found. The association of SNP NM_001526.4:c.922G > A was shown in the HCRTR2 gene, and NM_004898.4:c.213T> C in the CLOCK gene.
**Rainero et al., 2004**	Italy	Candidate gene	109 patients (96 episodic CH and 13 chronic) and 211 controls	Polymorphisms in *HCRT, HCRTR1, HCRTR2* genes were genotyped and analyzed by PCR-ARLS and PCR-ASO	*HCRT, HCRTR1, HCRTR2*	1246 G>A polymorphism of HCRTR2 gene was significantly associated with CH. Homozygosity for the G allele was associated with an increased disease risk (OR: 6.79, 95% CI, 2.25 to 22.99)
**Rainero et al., 2005a**	Italy	Candidate gene	109 CH patients, 211 controls	Genotyping of HFE gene for the C282Y and H63D mutations was performed using PCR and restriction enzyme digestion.	*HFE*	No significant association between C282Y or H63D polymorphisms and CH susceptibility. D63D genotype was associated with a later onset of CH ($p < 0.001$) when compared to H63H and H63D patients.
**Rainero et al., 2005b**	Italy	Candidate gene	107 patients, 210 controls	Genomic DNA was extracted using the QIAamp DNA Mini Kit, and genotyping of 3092 T°C Clock gene polymorphism was performed using PCR with Sdu I restriction enzyme digestion	*CLOCK*	No association found
**Rainero et al., 2008**	Italy	Candidate gene	109 CH patients, 211 healthy controls	Genotyping of HCRTR2 gene polymorphisms: rs10498801, rs3122156, rs9357855, rs2653342, rs3800539, and rs2653349 performed using PCR with digestion by restriction enzymes. Complete sequencing of the HCRTR2 gene in 11 CH patients. Haplotype analysis performed using PHASE 2.1 software.	*HCRTR2*	Three new polymorphisms of HCRTR2 gene (rs3122156T, rs2653342A, and rs2653349) were significantly associated with CH (p=.0053; p=.0082; p=.0002). Haplotype GTAAGG showed a higher frequency in cases (OR: 3.68; 95% CI: 1.85-7.67). No mutations found in sequencing.
**Rainero et al., 2010**	Italy	Candidate gene	110 patients (96 episodic CH and 14 chronic) and 203 controls	Patients and controls were genotyped for 2 bi-allelic SNPs of the ADH4 gene: rs1800759 and rs1126671 using the QIAamp DNA	*ADH4*	Genotype frequencies of the rs1126671 SNP resulted significantly different between patients and controls (χ^2 = 10.269, *P* = .006). AA genotype of

				mini Kit (QIAGEN). Allele, genotype, and haplotype frequencies of the examined SNPs were compared between cases and controls.		rs1126671 SNP associated with a 2 fold disease risk under a recessive model (OR = 2.33, 95% CI: 1.25-4.37).
**Ran et al., 2017**	Sweden	Candidate gene	542 CH, 581 controls	rs12668955 in ADCYAP1R1, rs1006417, an intergenic variant on chromosome 14q21 and one rare mutation, rs147564881 in MME were genotyped using TaqMan qPCR	*ADCYAP1R1, MME*	No association found
**Ran et al., 2018**	Sweden	Candidate gene and gene expression*	541 CH patients, 581 controls	Genotyping of rs1835740 in the MTDH gene and rs2651899 in the PRDM16 gene using TaqMan qPCR	*MTDH (metadherin), PRDM16*	rs1835740 in the MTDH gene significantly associated with CH (OR: 1.25; 95% CI: 1.01–1.53; p = 0.043). In patients with both CH and migraine, the association was stronger (p = 0.031; Chi-square = 6.964). No association found for the rs2651899 polymorphism in the PRDM16 gene. T allele of rs1835740 had a significant effect on the transcriptional activity of MTDH gene (*p* = 0.0318)
**Ran et al., 2019**	Sweden	Candidate gene and gene expression*	628 CH patients, 586 controls	Genotyping of rs1531394 in the ANO3 gene, ITGAL, PLCE1, and PCDHB6 using TaqMan qPCR and fibroblast gene expression analysis	*ANO3, ITGAL, PLCE1, PCDHB6*	rs1531394 in the ANO3 gene significantly associated with cluster headache (OR: 1.16; 95% CI: 1.01–1.34; p = 0.036). No significant associations found for ITGAL, PLCE1, or PCDHB6. No difference in the ANO3 gene expression in patients and controls
**Schürks et al., 2006**	Germany	Candidate gene	226 CH patients, 266 controls	Genomic DNA was extracted using the QiaAmp Mini DNA kit, amplified by PCR, and digested with Sau3AI for allele analysis via electrophoresis.	*HCRTR2*	Homozygous carriers of the G allele had a twofold increased risk for CH compared to heterozygous or homozygous carriers of the A allele (GG vs GA: OR 2.11, 95% CI 1.39 to 3.18, *p* = 0.0004; GG vs GA+AA: OR 1.97, 95% CI 1.32 to 2.92, *p* = 0.0007
**Schürks et al., 2011**	Germany	Candidate gene	147 patients, 599 controls	Genotypes of the *MTHFR* 677C>T polymorphism were detected by restriction fragment length polymorphism analysis	*MTHFR*	No association found

**Seibel et al., 1996**	Germany	Candidate gene	22 CH cases	Analysis of mitochondrial tRNA Leu(UUR) 3243A>G, 3250T>C, 3260A>G, and 3271A>G point mutations in 22 patients affected by CH (blood cells)	*mtRNALeu(UUR)*	No mutations found
**Shimomura et al., 1994**	Japan	Candidate gene	Case report	Analysis of mitochondrial tRNA Leu(UUR) 3243A>G point mutation in a patient affected by CH (blood cells)	*mtRNALeu(UUR)*	3243A>G point mutation detected in the gene
**Sjöstrand et al., 2001**	Sweden	Candidate gene	75 CH patients, 108 controls	An association analysis of an intragenic polymorphic (CA)n-repeat with marker D19S1150 and a (CAG)n-repeat in the 3'UTR region	*CACNA1A*	No association found
**Sjöstrand et al., 2002**	Sweden	Candidate gene	91 CH patients, 111 controls	Genotyping of microsatellite markers in the NOS1 (nNOS), NOS2A (iNOS), and NOS3 (eNOS) genes using PCR and fragment length analysis	*NOS1, NOS2A, NOS3*	No significant association between polymorphisms in NOS1, NOS2A, and NOS3 genes and CH susceptibility. The NOS2A 194-bp allele was significantly more common in controls (OR = 0.45; 95% CI: 0.25–0.82; p = 0.01), suggesting a potential protective effect.
**Summ et al., 2010**	Germany	Candidate gene	55 CH patients, 55 controls	Genotyping of SERPINA1 gene variants (F, M, S, Z alleles) using restriction fragment length polymorphism-polymerase chain reaction (RFLP-PCR)	SERPINA1	No association between CH and the common SERPINA1 genotypes. However, patients with non-MM genotypes (heterozygous or homozygous M allele carriers) had a higher frequency of CH attacks (p = 0.02).
**Weller et al., 2014**	Netherlands	Candidate gene and meta-analysis	575 patients, 874 controls	Genotyping of SNP rs2653349 (G1246A) was performed using a TaqMan assay and analyzed with LightCycler LC-480	*HCRTR2*	No association found
**Winsvold et al., 2023**		GWAS and meta-analysis	4777 CH, 31575 controls	Analyzed data from 10 European and 1 East Asian CH cohorts; those used in the 4 previous GWASs of CH and 5 additional cohorts, increasing the sample size for analysis 3.2-fold compared to the largest previous CH GWAS	*DUSP10, MERTK, FTCDNL1, FHL5, WNT2, PLCE1, and LRP1*	4 previously reported risk loci (DUSP10, MERTK, FTCDNL1, and FHL5), and 3 novel risk loci (WNT2, PLCE1, and LRP1) were identified

**Zarrilli et al., 2015**	Italy	Candidate gene	54 CH, 200 controls	The G1246A mutation of the HR2 gene, the T3092C mutation of the CLOCK gene, and ADH4 mutations rs1800759 and rs1126671 were analyzed by PCR followed by digestion with restriction enzymes	*HCRTR2, ADH4 and CLOCK*	Allele and genotype frequency of the rs1126671 (*p=0.03*) and rs1800759 (*p=0.03*) SNPs of the ADH4 gene resulted significantly. No associations found for HCRTR2 and CLOCK genes

* These studies also employ gene expression methodology

Supplementary Table 2. Summary of included functional genomic and pharmacogenomic studies

Reference	Country	Study Type	Sample	Methods	Genes Examined	Result highlights
**Costa et al., 2015**	Italy	Gene expression (microarray)	8 CH, 10 BD, and 10 controls	An exploratory microarray gene expression analysis in lymphoblasts from 8 CH and 10 BD I patients selected for positive response to lithium and 10 healthy controls.	*RBM3, NR1D1, TPH1*	RBM3 was the most significantly altered gene (3.17×10−13 in BD, 9.44×10−14 in CH). NR1D1 and TPH1 genes were also significantly altered both in CH and BD.
**Edvinsson et al., 2024**	Sweden	Gene expression	27 CH patients (episodic and chronic) and 30 controls	RT-qPCR was used to measure MERTK gene expression, and ELISA was employed to quantify MERTK ligands in serum.	*MERTK* gene	*MERTK* mRNA levels (t = -2.48, p-value = 0.018), and Gal-3 (p-value = 0.0022, p_c-value = 0.0067) were elevated in CH patients compared to controls.
**Eising et al., 2017**	Netherlands	Gene expression (RNA sequencing)	39 CH (19 episodic and 20 chronic CH) and 20 controls	RNA sequencing to identify differentially expressed genes and pathways in whole blood of patients with CH in comparison with headache-free controls	*N/A*	No associations were found with previously reported pathogenic mechanisms
**Gardiner et al., 1998**		Gene expression	20 patients with migraine (14 MWA and 6 MA), 12 CH patients and 8 controls	Levels of three G-protein mRNAs—Gsα, Giα, and Gα, were quantified in lymphocytes	Genes codifying for the α subunit Gs, Gq and Gi proteins	Consisted downregulation of Giα mRNA in all migraine groups whether quiescent or acute, with aura or without; Levels of Giα mRNA were significantly altered in CH patients compared to controls, but also differed significantly from those in migraine patients.
**Jennysdotter Olofsgård et al., 2024**	Sweden	Pharmacogenetic	545 CH patients genotyped for genetic variants: 409 triptan users and 109 non-users; clinical data from 893 patients	Patients were genotyped for five SNPs: *C12orf4* (rs1024905), Intergenic (rs6724624), *SLC6A4* (rs4795541), *GNB3* (rs5443), and *PRDM16* (rs2651899) using qPCR with TaqMan assays and PCR-RFLP.	*C12orf4, SLC6A4, GNB3, PRDM16*	rs1024905 in *C12orf4* was significantly associated with triptan non-usage in CH patients (p_c = 0.010). Patients with more effector variants were less likely to use triptans (p = 0.007). rs6724624 (Intergenic), rs4795541 (*SLC6A4*), rs5443 (*GNB3*), and rs2651899 (*PRDM16*), showed no significant association with triptan usage.
**Oliveira et al., 2024**	Portugal	Gene expression	50 CH patients and 58 matched controls	CLOCK gene expression measured by quantitative RT-PCR in peripheral blood over multiple	*CLOCK* gene	*CLOCK* expression fluctuated less in CH patients throughout the year compared to controls. Significant differences found

				seasons. RNA was isolated from blood samples collected at solstices and equinoxes. Expression levels were normalized to GAPDH.		in winter (p-value mean = 0.006283), spring (p-value mean = 0.000006), and summer (p-value mean = 0.000064), but not autumn.
**Petersen et al., 2023**	Norway, Denmark	Pharmacogenetic association	508 CH (166 chronic CH), 10000 controls	Genetic variation in CYP3A4 was genotyped on 30 ng genomic DNA using TaqMan drug metabolism genotyping assays for the following SNPs rs55785340, rs4986910, rs4987161 and rs35599367. PRS were calculated by the effect retrieved from a meta-analysis of the latest two GWAS on CH	*CYP3A4*	No evidence of associations between response to conventional abortive and preventive treatment and genetic risk of CH or functional variants of CYP3A4 was found. No genetic variants were associated with treatment response to triptans and verapamil in CH
**Schürks et al., 2007a**	Germany	Pharmacogenetic	184 CH patients	Genotyping of the G1246A polymorphism in the HCRTR2 gene using PCR and restriction fragment length polymorphism analysis	*HCRTR2*	No association found between the G1246A polymorphism and treatment response to triptans, oxygen, verapamil, or corticosteroids in CH patients.
**Schürks et al., 2007b**	Germany	Pharmacogenetic	231 CH patients	Genotyping of the GNB3 C825T polymorphism using PCR and restriction fragment length polymorphism analysis	*GNB3*	Heterozygous carriers of the GNB3 825T allele had a higher likelihood of responding to triptans (OR: 2.96; 95% CI: 1.34–6.56; p = 0.0074). No association was found for response to oxygen, verapamil, or corticosteroids.
**Schürks et al., 2014**	Germany	Pharmacogenetic	148 CH patients	Genotyping of 43-bp insdel (rs4795541) and A>G (rs25531) polymorphisms in the 5-HTTLPR promoter region using restriction fragment length polymorphism analysis	*SLC6A4 (5-HTTLPR)*	No association found between bi-allelic or tri-allelic 5-HTTLPR polymorphisms and triptan non-response in cluster headache patients.
**Sjöstrand et al., 2006**	Sweden	Gene expression	Microarray study: 3 patients; quantitative RT PCR: 6 CH and 14 controls	Microarray study and validation with quantitative RT-PCR. Blood samples collected in different phases of the disease	Affymetrix Human genome U133 2.0 Gene Chip set, 54.000 transcripts for 22.000 genes	S100 protein group genes, TNFRSF10C, ICAM3, RASEF, RARA, HLA-DQA1, DQB1, BIRC1 KCNJ15, CREB5, EIF5A, annexin A3 upregulation was shown. S100P upregulation confirmed with RT-PCR
**Steinberg et al., 2011**	Sweden	Gene expression	8 episodic CH patients, 16 healthy controls	Quantitative real-time PCR analysis of IL-2 gene expression	*IL-2*	Significantly increased IL-2 gene expression during active cluster headache period between attacks

			(median 9.9; IQR 6.2–10.3) compared to during attacks (median 2.8; IQR 0.7–3.2, p = 0.012), remission (median 1.6; IQR 0.9–1.8, p = 0.017), and controls (median 0.9; IQR 0.6–1.9, p = 0.0001).

Supplementary Table 3. Quality assessment of case-control studies

NEWCASTLE-OTTAWA SCALE CASE-CONTROL STUDIES (http://www.ohri.ca/programs/clinical_epidemiology/oxford.asp)										
**Study**	**Is the case definition adequate?**	**Representativeness of the cases**	**Selection of controls**	**Definition of controls**	**Comparability of cases and controls**	**Ascertainment of exposure**	**Same method of ascertainment for cases and controls**	**Nonresponse rate**	**Total**	**Quality rating**
**Bacchelli et al., 2016**	*	*	*	*	*	*	*		7	High
**Baumber et al., 2006**	*	*	*	*	*	*	*		7	High
**Cevoli et al., 2008**	*	*	*	*		*	*		6	Moderate
**Chen et al., 2022**	*	*	*	*	*	*	*		7	High
**Costa et al., 2015**	*	*	*	*	*	*	*		7	High
**Edvinsson et al., 2024**	*	*	*	*	*	*	*		7	High
**Eising et al., 2017**	*	*	*	*	*	*	*		7	High
**Fan et al., 2018**	*	*	*	*	*	*	*		7	High
**Fourier et al., 2016**	*	*	*	*	**	*	*	*	9	High
**Fourier et al., 2018**	*	*	*	*	*	*	*		7	High
**Fourier et al., 2019**	*	*	*	*	*	*	*		7	High
**Fourier et al., 2021**	*	*	*	*	*	*	*	*	8	High
**Gardiner et al., 1998**	*	*	*	*		*	*		6	Moderate
**Harder et al., 2021**	*	*	*	*	**	*	*		8	High
**Jennysdotter et al., 2021**	*	*	*	*	*	*	*		7	High
**Jennysdotter et al., 2023**	*	*	*	*	*	*	*		7	High
**Jennysdotter et al., 2024**	*	*	*	*	**	*	*		8	High
**O'Connor et al., 2021**	*	*	*	*	*	*	*		7	High
**Ofte et al., 2016**	*	*	*	*	**	*	*	*	7	High

**Oliviera et al., 2024**	*	*	*	*	**	*	*		8	High
**Papasavva et al., 2020**	*	*	*	*		*	*		6	Moderate
**Papasavva et al., 2022a**	*	*	*	*	**	*	*		8	High
**Papasavva et al., 2022b**	*	*	*	*	**	*	*		8	High
**Petersen et al., 2024**	*	*	*	*	**	*	*	*	9	High
**Rainero et al., 2004**	*	*	*	*		*	*		6	Moderate
**Rainero et al., 2005a**	*	*	*	*		*	*		6	Moderate
**Rainero et al., 2005b**	*	*	*	*		*	*		6	Moderate
**Rainero et al., 2008**	*	*	*	*	*	*	*		7	High
**Rainero et al., 2010**	*	*	*	*	*	*	*		7	High
**Ran et al., 2017**	*	*	*	*		*	*		6	Moderate
**Ran et al., 2018**	*	*	*	*	*	*	*		7	High
**Ran et al., 2019**	*	*	*	*	*	*	*		7	High
**Schürks et al., 2006**	*	*	*	*		*	*		6	Moderate
**Schürks et al., 2011**	*	*	*	*	*	*	*		7	High
**Sjöstrand et al., 2001**	*	*	*	*		*	*		6	Moderate
**Sjöstrand et al., 2002**	*	*	*	*		*	*		6	Moderate
**Sjöstrand et al., 2006**	*	*	*	*		*	*		6	Moderate
**Steinberg et al., 2011**	*	*	*	*		*	*		7	Moderate
**Summ et al., 2010**	*	*	*	*	*	*	*		6	Moderate
**Weller et al., 2014**	*	*	*	*	*	*	*		7	High
**Winswold et al., 2023**	*	*	*	*	**	*	*		8	High
**Zarilli et al., 2015**	*	*	*	*		*	*		6	Moderate

Supplementary Table 4. Quality assessment of observational cohort and cross-sectional studies

Study	Q1	Q2	Q3	Q4	Q5	Q6	Q7	Q8	Q9	Q10	Q11	Q12	Q13	Q14	Quality
**Cortelli et al., 1995**	Y	Y	NR	NR	N	Y	Y	NA	Y	N	NR	NA	NA	NR	Poor
**Haan et al., 2001**	Y	Y	Y	Y	NA	Y	Y	NA	Y	N	Y	Y	NA	NR	Fair
**Petersen et al., 2023**	Y	Y	Y	Y	Y	Y	Y	Y	Y	N	Y	NA	NA	Y	Good
**Popescu et al., 2023**	Y	Y	NA	Y	N	Y	Y	NA	Y	N	Y	NA	NA	N	Fair
**Schürks et al., 2007a**	Y	Y	Y	Y	N	Y	Y	Y	Y	N	Y	NA	NA	Y	Good
**Schürks et al., 2007b**	Y	Y	Y	Y	N	Y	Y	Y	Y	N	Y	NA	NA	Y	Good
**Schürks et al., 2014**	Y	Y	Y	Y	N	Y	Y	Y	Y	N	Y	NA	NA	Y	Good
**Seibel et al., 1996**	Y	Y	NR	Y	N	Y	Y	NA	Y	N	Y	NA	NA	NR	Poor
**Shimomura et al., 1994**	N	Y	NA	NA	NA	Y	Y	NA	Y	N	Y	NA	NA	N	Poor

Quality of included studies was assessed using the National Institutes of Health (NIH) Quality Assessment tool for Observational Cohort and Cross-Sectional Studies. (https://www.nhlbi.nih.gov/health-topics/study-quality-assessment-tools) **Q1.** Was the research question or objective in this paper clearly stated? **Q2**. Was the study population clearly specified and defined? **Q3**. Was the participation rate of eligible persons at least 50%? **Q4**. Were all the subjects selected or recruited from the same or similar populations (including the same time period)? Were inclusion and exclusion criteria for being in the study prespecified and applied uniformly to all participants? **Q5**. Was a sample size justification, power description, or variance and effect estimates provided? **Q6**. For the analyses in this paper, were the exposure(s) of interest measured prior to the outcome(s) being measured? **Q7**. Was the timeframe sufficient so that one could reasonably expect to see an association between exposure and outcome if it existed? **Q8**. For exposures that can vary in amount or level, did the study examine different levels of the exposure as related to the outcome (e.g., categories of exposure, or exposure measured as continuous variable)? **Q9**. Were the exposure measures (independent variables) clearly defined, valid, reliable, and implemented consistently across all study participants? **Q10**. Was the exposure(s) assessed more than once over time? **Q11**. Were the outcome measures (dependent variables) clearly defined, valid, reliable, and implemented consistently across all study participants? **Q12**. Were the outcome assessors blinded to the exposure status of participants? **Q13**. Was loss to follow-up after baseline 20% or less? **Q14**. Were key potential confounding variables measured and adjusted statistically for their impact on the relationship between exposure(s) and outcome(s)? **CD,** cannot be determined; **NA,** not applicable; **NR,** not reported; **N,** no; **Y,** yes. **Overall quality:** poor, fair, or good.
